# What is the impact of primary care model type on specialist referral rates? A cross-sectional study

**DOI:** 10.1186/1471-2296-15-22

**Published:** 2014-02-03

**Authors:** Clare Liddy, Jatinderpreet Singh, Ryan Kelly, Simone Dahrouge, Monica Taljaard, Jamie Younger

**Affiliations:** 1 C.T. Lamont Primary Health Care Research Centre, Bruyère Research Institute, 43 Bruyère St. Room 369Y, Ottawa, Ontario K1N 5C8, Canada; 2 Department of Family Medicine, University of Ottawa, 43 Bruyère St. Room 369Y, Ottawa, Ontario K1N 5C8, Canada; 3 Institute for Clinical Evaluative Sciences, Ottawa, Ontario, Canada; 4 Clinical Epidemiology Program, Ottawa Hospital Research Institute, Ottawa, Ontario, Canada; 5 Department of Epidemiology and Community Medicine, University of Ottawa, Ottawa, Ontario, Canada

**Keywords:** Primary care, Specialist referral, Capitation, Primary care model

## Abstract

**Background:**

Several new primary care models have been implemented in Ontario, Canada over the past two decades. These practice models differ in team structure, physician remuneration, and group size. Few studies have examined the impact of these models on specialist referrals. We compared specialist referral rates amongst three primary care models: 1) Enhanced Fee-for-service, 2) Capitation- Non-Interdisciplinary (CAP-NI), 3) Capitation – Interdisciplinary (CAP-I).

**Methods:**

We conducted a cross-sectional study using health administrative data from primary care practices in Ontario from April 1st, 2008 to March 31st, 2010. The analysis included all family physicians providing comprehensive care in one of the three models, had at least 100 patients, and did not have a prolonged absence (eight consecutive weeks). The primary outcome was referral rate (# of referrals to all medical specialties/1000 patients/year). A multivariable clustered Poisson regression analysis was used to compare referral rates between models while adjusting for provider (sex, years since graduation, foreign trained, time in current model) and patient (age, sex, income, rurality, health status) characteristics.

**Results:**

Fee-for-service had a significantly lower adjusted referral rate (676, 95% CI: 666-687) than the CAP-NI (719, 95% confidence interval (CI): 705-734) and CAP-I (694, 95% CI: 681-707) models and the interdisciplinary CAP-I group had a 3.5% lower referral rate than the CAP-NI group (RR = 0.965, 95% CI: 0.943-0.987, p = 0.002). Female and Canadian-trained physicians referred more often, while female, older, sicker and urban patients were more likely to be referred.

**Conclusions:**

Primary care model is significantly associated with referral rate. On a study population level, these differences equate to 111,059 and 37,391 fewer referrals by fee-for-service versus CAP-NI and CAP-I, respectively – a difference of $22.3 million in initial referral appointment costs. Whether a lower rate of referral is more appropriate or not is not known and requires further investigation. Physician remuneration and team structure likely account for the differences; however, further investigation is also required to better understand whether other organizational factors associated with primary care model also impact referral.

## Background

A key component of a primary care physician’s role involves acting as a gatekeeper to medical specialists and other health resources to ensure that patients receive appropriate and timely care. Studying referrals is of importance to policy makers and healthcare professionals, due to their significant effects on healthcare costs, quality of care, patient safety, and access to care [1,2]. As such, having a clear understanding of how system level reforms impact specialist referrals is important.

Over the past two decades, many nations worldwide have initiated reforms to improve their delivery of primary health care. In Canada, the healthcare system is funded through public funds and is freely available to the population with the administrative responsibility of providing the healthcare services devolved to the individual provinces. Several provinces have recently redesigned their primary health care systems with the goal of improving patient access, preventive care, chronic disease care, and coordination with other health services [3].

In Ontario, Canada’s most populous province, the primary focus of reforms has been on the development of interdisciplinary health care teams and on shifting away from the traditional fee-for-service (FFS) physician remuneration model to a capitated payment approach [4]. Through capitation, providers receive a sex and age adjusted fixed payment per patient, which is independent of the number of services performed. Two types of capitation models have emerged with one being associated with funded interdisciplinary teams, and the second one without these teams. The primary care physicians were able to choose and apply to participate in either model, although limitations on the team funding restricted the total number of capitation interdisciplinary teams approved each year in Ontario. These models developed historically and now involve three quarters of the Ontario population. This is a natural experimental environment to understand the impact of primary care reforms on the health system as a whole. Previous studies comparing the delivery of care across the different models in Ontario have demonstrated differences in areas such as health promotion, chronic disease care, and health equity [5-7]. Few studies have examined the impact of different models on other health care sectors such as specialty care.

The aim of this study is to examine differences in specialist referral rates amongst three main primary care models in Ontario, Canada: 1) Enhanced FFS, 2) Capitation- Non-Interdisciplinary (CAP-NI), and 3) Capitation – Interdisciplinary (CAP-I) . We hypothesized that 1) the two CAP models would have higher referrals than the FFS because of the reduced financial compensation for services delivered, and that 2) CAP-I practices would have lower referral rates than the CAP-NI group, as providers in this model have greater onsite resources and opportunity for collaboration than non-interdisciplinary practices

## Method

### Study design

We conducted a cross-sectional study using healthcare administrative data collected from April 1st, 2008 to March 31st, 2010 to compare patient referral rates between three different primary care models in Ontario, Canada.

### Models

The three models investigated in this study involve approximately three quarters of both the physician and patient populations in the province of Ontario [8]. They are all physician led in terms of governance, and have after hours access requirements .The key differences between the three primary care models examined in this study are physician remuneration and team structure. In the Enhanced fee-for-service (FFS) model, physicians receive the majority of their payment through fee for service billing, but also receive incentive and premium payments for patient enrolment, health promotion activities, and for the management of certain conditions (e.g., diabetes). Practices in this group have a traditional structure; that is the physicians working alone or sharing office space with limited administrative staff and/or nurses funded by the physicians.

In the Capitation Non-Interdisciplinary (CAP-NI) model, physicians are paid primarily through capitation and typically work in a traditional practice structure. Under capitation, physicians receive a fixed base payment (adjusted for age and sex) for each enrolled patient and also receive 15% of the usual FFS billing and incentives for the delivery of specific services (e.g., diabetes care, smoking cessation counselling). Services outside the basket of services included under capitation are billed at 100%.

The Capitation Interdisciplinary (CAP-I) model are also capitation based, but includes large interdisciplinary health care teams including family physicians, nurses, and other health professionals such as dieticians, nurse practitioners, pharmacists, and social workers. The additional team members are funded by the government. Both capitation models encourage a maximum patient roster size of 2400 patients per full time physician by reducing the capitation payment by half for patients rostered beyond 2400.

### Population

Access to specialist care in Ontario requires a referral by a primary care physician. We examined the referral patterns of all active primary care physicians who were providing comprehensive care and belonged to one of the aforementioned models between April 1st, 2008 and March 31st, 2010. Physicians were excluded if they had a prolonged period of absence (i.e., eight or more consecutive weeks of inactivity), were not providing comprehensive care (i.e., having not billed 8 of the 18 standard primary care fee schedule codes), or had less than 100 patients.

### Data sources

Data for this study were obtained from healthcare administrative databases housed at the Institute for Clinical Evaluative Sciences (http://www.ices.on.ca/). Information regarding referrals was obtained from the Ontario Health Insurance Plan database, which contains billing claims for all ambulatory visits with physicians across the province in conjunction with the Corporate Provider Database to identify the speciality of the billing physician, the model in which they practice and their socio-demographic information. The Client Agency Program Enrolment Database was used to assign patients to physicians to whom they are enrolled. Approximately 15% of patients were not officially enrolled with a family physician. These were attributed to the family physician from whom they received the largest amount (dollar value) of services in the previous two years. The Registered Persons Database was used for patient demographic information (e.g., age, sex, etc). The patient postal code and census data from Statistics Canada was used to assign patients the income quintiles and rurality of their neighbourhood. We used Aggregated Diagnosis Groups (ADGs), which is part of the Johns Hopkins Adjusted Clinical Group case-mix system that measures patient comorbidity and morbidity [9].

### Outcome

The primary outcome was the patient referral rate expressed as: number of referrals to all medical specialties per 1000 patients per year. For each referral, we also collected data on the type of medical specialty to which the patient was referred. Here we report only on the aggregate referrals to any medical specialty.

The number of referrals for each patient was examined over a two year timeframe (April 1^st^, 2008 to March 31^st^, 2010). If a patient was referred to the same specialist multiple times during the timeframe, this was only counted as a single referral. This was done to avoid counting repeat referrals for the same patient complaint. On the other hand, if a patient was referred to two physicians of the same specialty during the study timeframe, this was counted as two referrals.

### Data analysis

Descriptive statistics were generated to describe provider and patient characteristics overall and by model type. Multivariable Poisson regression analysis was used to compare referral rates between the model types. The dependent variable was specified as the number of referrals for each patient over the two year study period. The primary independent variable, primary care model type, was specified as a three level categorical variable (FFS, CAP-NI, CAP-I). We initially ran an unadjusted regression (Model A) model and then adjusted referral rates for provider and patient characteristics by including these variables as covariates in the regression model. We first adjusted for patient characteristics only (age, sex, income, rurality, health status) (Model B). In a subsequent analysis, four provider characteristics were added to the model (sex, years since graduation, foreign trained, time in current model) (Model C). These characteristics were chosen as they have been shown to impact practice patterns [10,11]. All regression models accounted for clustering of patients by PCPs using Generalized Estimating Equations (GEE). Pairwise comparisons between model types were judged at the 0.017 Bonferroni-adjusted level to account for multiplicity and to maintain the familywise error rate at 5%. Estimated regression coefficients were expressed as Relative Rates (RRs) with 95% confidence intervals (CI). Least square mean estimates of referral rates for each model type were calculated by setting covariate values equal to their population mean values. Analyses were conducted using SAS, Version 9.3, SAS Institute Inc.

## Results

Six thousand three hundred and seventy providers caring for slightly over 10 million individuals were included in the analysis, with 53% cared for in the FFS practices, 26% in CAP-NI and 20% in CAP-I practices Over the two year examination timeframe, there were a total of 16,286,998 referrals across all medical specialties. The majority of specialist referrals were for diagnostic radiology (40.0%), followed by cardiology (8.7%), internal medicine (4.1%), and general surgery (4.1%).

Table 1 presents provider and patient characteristics for each model. In general, there were some differences in the year since graduation across models, and a higher percentage of foreign trained physicians in FFS practices. The patient population in FFS practices also tended to be sicker and a greater proportion resided in urban areas.

**Table 1:** Provider and patient level characteristics by model

**Characteristic**	**Primary care model type**			**All n = 6370**
	**FFS**	**CAP-NI**	**CAP-I**	
	**n = 3357**	**n = 1591**	**n = 1 422**	
**Physician level**				
Female (n, %)	1285 (38.3)	588 (37.0)	570 (40.1)	2443 (38.4)
Years since graduation (mean, SD)	26.4 (11.0)	25.1 (10.5)	23.7 (10.6)	25.5 (10.8)
Foreign trained (n, %)	1125 (33.6)	243 (15.3)	206 (14.5)	1574 (24.8)
Years practicing in current model (mean, SD)	4.0 (1.8)	1.7 (1.3)	2.4 (2.2)	3.1 (2.1)
**Patient level**	n = 5 390 238	n = 2 573 253	n = 2 083 383	n = 10 046 874
Female (n,%)	2 821 266 (52.3)	1 345 330 (52.3)	1 104 388 (53.0)	5 270 984 (52.5)
Age in years (n, %)				
0–19	1 215 419 (22.6)	570 824 (22.2)	478 242 (23.0)	2 264 485 (22.5)
20–39	1 463 203 (27.2)	620 080 (24.1)	499 797 (24.0)	2 583 080 (25.7)
40–59	1 674 815 (31.1)	796 341 (31.0)	628 284 (30.2)	3 099 440 (30.9)
60–79	843 309 (15.7)	465 495 (18.1)	379 943 (18.2)	1 688 747 (16.8)
80+	193 492 (3.6)	120 513 (4.7)	97 117 (4.7)	411 122 (4.1)
Aggregated diagnosis group (n, %)				
0	585 650 (10.9)	356 024 (13.8)	336 593 (16.2)	1 278 267 (12.7)
1	3 283 238 (60.9)	1 649 720 (64.1)	1 340 816 (64.4)	6 273 774 (62. 5)
2	1 386 818 (25.7)	521 824 (20.3)	373 666 (17.9)	2 282 308 (22.7)
3	134 532 (2.5)	45 685 (1.8)	32 308 (1.6)	212 525 (2.1)
Income quintile (n, %)				
1 (low)	1 037 373 (19.4)	390 390 (15.3)	362 374 (17.5)	1 790 137 (17.9)
2	1 080 224 (20.2)	454 207 (17.8)	400 160 (19.3)	1 934 591 (19.4)
3	1 110 734 (20.7)	503 332 (19.7)	413 768 (20.0)	2 027 834 (20.3)
4	1 131 680 (21.1)	574 193 (22.5)	451 132 (21.8)	2 157 005 (21.6)
5 (High)	999 066 (18.6)	635 649 (24.9)	442 822 (21.4)	2 077 537 (20.8)
Rurality index (n, %)				
≥45	92 378 (1.7)	92 125 (3.6)	202 923 (9.9)	387 426 (3.9)
10–44	760 940 (14.2)	811 197 (31.8)	763 140 (37.1)	2 335 277 (23.4)
≤10	4 499 476 (84.1)	1 651 700 (64.7)	1 092 498 (53.1)	7 243 674 (72.7)

The results of the multivariable regression model, adjusting for both patient and provider characteristics are presented in Table 2. Few changes were observed in regression coefficients for patient level characteristics when adding provider characteristics to the model; results are therefore presented for the full multivariable model only. All patient and provider level characteristics (including model type) were significantly related to the referral rate. Referral rates were higher for providers who were female, had more years in practice and for those who were trained in Canada. In terms of patient characteristics, those that were older, sicker, female, and resided in urban areas had higher referral rates.

**Table 2:** Patient and provider adjusted relative risk* (RR) from the multivariable regression model

**Independent variable**	**Levels**	**Relative risk (RR)**	**95% confidence interval for RR**	**P-value**
Primary care model	CAP-I	0.965	0.943–0.987	0.0021
	FFS	0.940	0.917–0.963	<.0001
	CAP-NI	1.000	-	.
**Patient characteristics**				
Health status (ADG)	3 (Very sick)	8.464	8.358–8.571	<.0001
	2	5.846	5.787–5.906	<.0001
	1	3.020	2.996–3.043	<.0001
	0 (Healthy)	1.000	-	.
Income quintile	5 (high)	1.041	1.038–1.044	<.0001
	4	1.041	1.038–1.044	<.0001
	3	1.031	1.028–1.034	<.0001
	2	1.020	1.018–1.023	<.0001
	1 (low)	1.000	-	.
Rurality	Rural	0.935	0.925–0.945	<.0001
	Non-major urban centre	0.990	0.984–0.995	0.0001
	Major urban centre	1.000	1.000	.
Patient age	0-21	3.591	3.558–3.623	<.0001
	22-40	2.986	2.962–3.011	<.0001
	41-56	1.895	1.883–1.908	<.0001
	57+	1.000	-	.
Patient sex	Female vs. male	1.172	1.169–1.175	<.0001
**Physician characteristics**				
Physician sex	Female vs. male	1.145	1.124–1.165	<.0001
Year of graduation		1.003	1.002–1.004	<.0001
Foreign trained	Foreign vs. local	0.926	0.906–0.946	<.0001
Time in model		1.001	1.001–1.001	<.0001

*A relative risk greater than one indicates that the patient group was more likely to be referred than the specified reference group, whereas a value below one indicates that they are less likely to be referred relative to the reference group.

**Figure 1:**
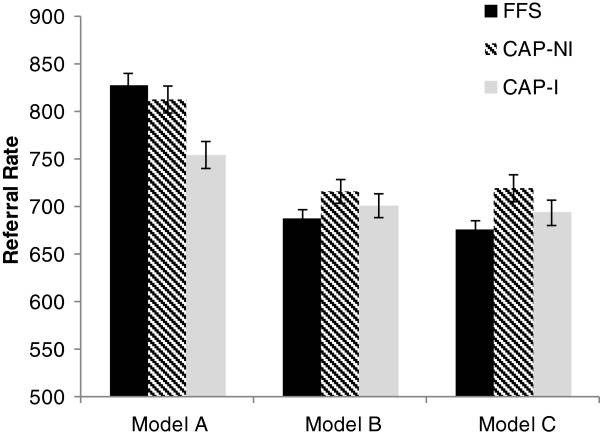
Unadjusted and adjusted least square mean estimates of referral rates by primary care model.

Figure 1 presents the least square mean estimates of the referral rates in the three model types. The CAP-I model had a lower unadjusted referral rate (755, 95% CI: 741-770 ) than both the CAP-NI (814, 95% CI: 799-827) and the FFS (827, 95% CI: 814-841 CAP-I) models. However, after adjusting for relevant patient and provider characteristics, FFS practices had the lowest referral rate (676, 95% CI: 666-687, p < 0.0001) when compared to the other two models (CAP-NI: 719, 95% CI: 705-734, p < 0.0001; CAP-I: 694, 95% CI: 681-707, p < 0.0001).

The two CAP models were also significantly different (p = 0.002). Pairwise comparisons of the adjusted referral rates [Table 3] revealed that the interdisciplinary CAP-I group had a 3.5% lower referral rate than the CAP-NI group (RR = 0.965, 95% CI: 0.943-0.987, p = 0.002).

**Table 3:** Unadjusted and adjusted pairwise comparisons of referral rates between primary care models

**Regression model**	**Comparison of primary care models**		**Relative difference in rates**	**95% confidence interval**	**P-value**
Model A (Unadjusted)	CAP-I	FFS	0.913	0.890–0.936	<0.0001
	CAP-I	CAP-NI	0.929	0.905–0.953	<0.0001
	FFS	CAP-NI	1.018	0.994–1.042	0.15
Model B (Patient adjusted)	CAP-I	FFS	1.021	1.000–1.043	0.049
	CAP-I	CAP-NI	0.980	0.958–1.002	0.071
	FFS	CAP-NI	0.959	0.940–0.979	<0.0001
Model C (Patient and provider adjusted)	CAP-I	FFS	1.027	1.004–1.050	0.02
	CAP-I	CAP-NI	0.965	0.943–0.987	0.002
	FFS	CAP-NI	0.940	0.917–0.963	<0.0001

## Discussion

This study demonstrates significant differences in specialist referral patterns between primary care models. After accounting for patient and provider characteristics, physicians practicing in the FFS model had a lower referral rate than the physicians in the two capitated models (i.e., CAP-NI and CAP-I), while physicians in the interdisciplinary CAP-I model had a lower referral rate than those in the non-interdisciplinary CAP-NI model.

On a study population level, the observed differences in referral rates equate to 111,059 and 37,391 fewer referrals by FFS versus CAP-NI and CAP-I, respectively – a total difference of $22.3 million in initial referral appointment costs (assuming that the average cost of a referral is $150 [12]). The observed referral cost difference between CAP-I and CAP-NI is $ 9.9 million. We expect this cost difference to increase when including other costs that are frequently incurred during or following a typical referral (e.g., patient travel costs, time off work, repeat diagnostic testing, follow up specialist visits etc.).

These results confirm our hypotheses which were premised on 1. Remuneration influencing physician behaviour, and 2. Team structure influencing capacity. Physicians that are paid primarily through a fee-for-service approach may have an incentive to bring patients in multiple times to bill for more services instead of referring out to a specialist. On the other hand, in a capitation-based payment model, physicians are given a fixed lump-sum payment for each patient regardless of the number of services they provide and may be more inclined to pass on the treatment of their patients to specialists. This is in line with findings from several European-based primary care studies, which found that FFS was associated with a 9-12% lower referral rate than capitation or salary payment models [13-16].

Physicians practicing in the interprofessional capitation model had a lower referral rate than those working in the CAP-NI, potentially because the presence of allied health professionals such as a social worker, nurse educator and pharmacist gives greater capacity for care afforded to the doctor by the team structure that results in a lower pressure to offload complex, time consuming patients. For example a team comprised of a nurse and a pharmacist can support insulin initiation for a poorly controlled patient with diabetes thereby avoiding the referral to a specialist endocrinology clinic. A team with a social worker can help provide mental health support to patients thereby avoiding referral to a psychiatrist.Evidence examining the impact of interdisciplinary primary care practices on specialist referrals is limited and mixed, with certain studies showing no impact while others have shown a decrease in referral rates [17,18]. Thus,there is a need for ongoing research in this area to understand the impact of team based care from both an access and an economic perspective. Patient characteristics had the largest impact on referral rates; inversing the observed associations between referral rate and model. Patients that were older, sicker, female and urban residents had higher referral rates, a finding which is consistent with studies that have been conducted internationally and in Canada [1,10,11,19]. Females were 17.2% more likely to have a referral than males; however, this is likely due in most part to referrals made to obstetrics/gynecology. In the case of rurality, urban residents were 6.5% more likely to have a referral than those in rural areas. Primary care physicians in rural areas likely provide a select range of specialty services due to the shortage of local specialists and travel challenges for patients within these communities [10,20].

In addition, gender of the physician had a significant impact on referral rates with female physicians more likely to refer patients compared to male doctors. The reason for this difference is unclear and likely involves multiple factors ranging from differences in time spent with the patient resulting in more indepth examinations leading to referral, or to differences in risk tolerance [21]. Our results are consistent with a previous study in Ontario [10] and a recent study from the Netherlands [21]. This study was limited due to low power with the total number of physicians being only 44 with 14 female physicians in the sample. Other studies have not found this same effect of physician gender on referral patterns and suggest it may be related to other organizational aspects of the practice that differ between men and women [22,23]. Nonetheless, as there is increasing proportion of women in the physician workforce, differences in referral rates could have major implications for health system planning and resources. Future studies should include physician gender as a variable as earlier research on the variability in referral rates did not commonly include physician gender in the analysis.

### Study limitations

The analyses conducted in this study relied on health administrative databases which have certain limitations based on availability of the data. Salaried physicians who work in community health centres in Ontario do not bill to OHIP, and thus, we could not extract referral data on these physicians and thus they were excluded from this study. That being said, CHCs in Ontario only treat 0.9% of the total population, and the services and patient population they treat are unique in comparison to the other models [24]. Another limitation of this study is that we have no data on the appropriateness of the referrals made by each model. Based on the data in this study, we are unable to conclusively state whether having a higher referral rate is a positive or negative outcome in regards to patient safety or resource utilization. Lastly, although both physician remuneration and team structure likely contribute to the observed differences in referral rates, we cannot exclude the possibility that other, unmeasured factors confound that relationship.

## Conclusions

These findings demonstrate that there is a significant association between primary care model type and referral rates. Whether a lower rate of referral is more appropriate or not is not known and requires further investigation. Our findings suggest that physician remuneration and practice team structure may account for the differences in models; however, further investigation is required to better understand whether other factors associated with primary care model also impact referral rates. This study is one of few to comprehensively examine the association between primary care model type and referral rates on a large population basis. This study provides data which will help policy makers understand the impact of recent primary care reforms on speciality care and will also provide support in planning and projecting future referral initiatives and their impact on health care costs.

## Abbreviations

ADG: Aggregated diagnosis group; CAPE: Client agency program enrolment; CAP-I: Capitation – interdisciplinary; CAP-NI: Capitation - non-interdisciplinary; CI: Confidence interval; FFS: Fee-for-service; ICES: Institute for clinical evaluative sciences; IPDB: ICES physician database; OHIP: Ontario health insurance plan; PCP: Primary care physician; RR: Rate ratio.

## Competing interests

The authors declare that they have no competing interests.

## Authors’ contributions

CL and JS conceived the idea for this study. All authors contributed to the design of the analysis plan, while MT and JY conducted the data analysis. All authors critically reviewed and approved the final manuscript.

## Pre-publication history

The pre-publication history for this paper can be accessed here:

http://www.biomedcentral.com/1471-2296/15/22/prepub
