# Erratum: What is the impact of primary care model type on specialist referral rates? A cross-sectional study

**DOI:** 10.1186/s12875-015-0260-7

**Published:** 2015-05-24

**Authors:** Clare Liddy, Jatinderpreet Singh, Ryan Kelly, Simone Dahrouge, Monica Taljaard, Jamie Younger

**Affiliations:** C.T. Lamont Primary Health Care Research Centre, Bruyère Research Institute, 43 Bruyère St. Room 369Y, Ottawa, ON Canada; Department of Family Medicine, University of Ottawa, Ottawa, ON Canada; Institute for Clinical Evaluative Sciences, Ottawa, ON Canada; Clinical Epidemiology Program, Ottawa Hospital Research Institute, Ottawa, ON Canada; Department of Epidemiology and Community Medicine, University of Ottawa, Ottawa, ON Canada

## Erratum

After publication of this research article [1], we noted an error in Table 2. The values reported under Patient Age had been erroneously inverted, meaning age range “0-21” displayed the value for “57+” and vice versa, and “22-40” displayed the value for “41-56” and vice-versa. This error has been corrected (please see the revised version of Table 2 below). We apologise for any inconvenience.

**Table 2:** **Patient and provider adjusted rate ratios (RR) from the multivariable regression model**

**Independent variable**	**Levels**	**Rate Ratio (RR)**	**95% Confidence interval for RR**	**P-Value**
Primary Care Model	CAP-I	0.965	0.943-0.987	0.0021
	FFS	0.940	0.917-0.963	<.0001
	CAP-NI	1.000	-	.
**Patient characteristics**				
Health Status (ADG)	3 (Very Sick)	8.464	8.358-8.571	<.0001
	2	5.846	5.787-5.906	<.0001
	1	3.020	2.996-3.043	<.0001
	0 (healthy)	1.000	-	.
Income Quintile	5 (High)	1.041	1.038-1.044	<.0001
	4	1.041	1.038-1.044	<.0001
	3	1.031	1.028-1.034	<.0001
	2	1.020	1.018-1.023	<.0001
	1 (low)	1.000	-	.
Rurality	Rural	0.935	0.925-0.945	<.0001
	Non-major urban centre	0.990	0.984-0.995	0.0001
	Major urban centre	1.000	1.000	.
Patient Age	57+	3.591	3.558-3.623	<.0001
	41-56	2.986	2.962-3.011	<.0001
	22-40	1.895	1.883-1.908	<.0001
	0-21	1.000	-	.
Patient Sex	Female vs. male	1.172	1.169-1.175	<.0001
**Physician Characteristics**				
Physician Sex	Female vs. male	1.145	1.124-1.165	<.0001
Year of Graduation		1.003	1.002-1.004	<.0001
Foreign Trained	Foreign vs. local	0.926	0.906-0.946	<.0001
Time in Model		1.001	1.001-1.001	<.0001
